# Sero-Therapy in Tuberculosis

**Published:** 1896-10

**Authors:** 


### Abstracts and Selections.

**Sero-Therapy in Tuberculosis.**

Abstract of report to American Medical Association, appearing in *Journal of the American Medical Association.*—Paul Paquin, M. D., of St. Louis, member of the State Board of Health of Missouri, delivered before the American Medical Association an interesting report of cases by various physicians on treatment of tuberculosis by serum, of which we give an abstract.

The report made the American Medical Association by Dr. Paquin in 1895, on the value of serum in tuberculosis, is confirmed, and a number of new clinical reports, giving recoveries and referring to favorable reports of more than sixty physicians in America using the serum, and the recent work of Maragliano, of Genoa; Behring, of Berlin; Roux, of Paris; Winternich, Foa, et. al., is cited in further confirmation.

The close similarity in production and in action between the serum for tuberculosis and the serum for diphtheria is pointed out, with such differences as are due to the life and habits of the differing germs.

Phagocytosis is declared the basis of the defense of the body from micro-organic diseases, and to be the chief power of the antitoxines.

Inoculating the horse repeatedly with increasing quantities of a certain toxine, nature produces the corresponding antitoxine. This explains the toleration of the animal to subsequent increasing doses of toxine. The antitoxine, in a physiologic way, neutralizes the toxine; and thus prepared for man, neutralizes the toxine generated in his body, controls the attendant irritation of adjacent tissue, whereby the medium for germ growth is obtained, and enables nature to throw off and destroy the germ itself. The drain of this vital chemistry is put on the powerful system of the horse, and the antitoxine delivered in the serum to man without any drain on the human system, which would attend the use of tuberculine (a toxine) in man.

The serum effect must not, however, be over estimated, expected to replace lost tissue, restore fatal lesions, or revive the moribund.

It is of radical and extreme importance to diagnose tuberculosis at the earliest possible moment before too grave mixed infection takes place, or lesions necessarily fatal occur.

A large number of reports are cited, but omitted here.

---

### Selected Formulæ.

---

PRURITUS OF THE VULVA.—In cases that are not parasitic, says the *Independance Medicale*, M. Mussy advises the following applications:

℞ Finely powdered starch...........300 grains

Bismuth subnitrate, } of each, 15 "

Potassium bromide, }

Calomel .................................... 8 "
Powdered belladonna............. 3 "

M. To be applied twice a day. It is said to give most instant relief.

When the itching affects the inner surface of the mucous membrane, it is preferable to prescribe the following:

℞ Infusion of mallow flowers...... 1 quart.
Cherry-laurel water...............750 grains.
Borax ..................................150 "

M. To be used as an injection twice a day. After each injection, the parts are to be smeared with an ointment of one part of chloroform to ten parts of vaseline.

Bernheim (*Vienna Clinical Formulary; ibid.*) prefers cocaine to chloroform, and gives the following formulæ:

(1) ℞ Emollient ointment...............100 parts.
Cocaine hydrochloride........... 1 part.
M.

(2) ℞ Pure carbolic acid................ 1 part.
Water ................................ 20 parts.
M. Sig.: To be painted on.

The Insomnia of Neurasthenia. — Monin (*Independance med.*, July 1st) says the following draught is well borne for a long time:

℞ Paraldehyde ........................ 38 grains.
Fluid extract of piscidia.......... 75 "
Syrup of cherry-laurel...........750 "

M. S.: The whole to be taken at once in a cup of orange-flower water.—*N. Y. Med. Journal.*

---

**Preparation of Gauze Dressing** (*New York Medical Journal*).—Dr. Martenson gives the following directions: Rolls of cheesecloth about thirty yards in length are folded and placed in jars. On these the following solutions are poured, depending upon what kind of gauze it is desired to produce. Carbolized gauze, five per cent.:

℞ Colophene.............................. 50 parts.
Castor oil.................................. 15 "
Carbolic acid.............................. 28 "
Alcohol, 90°..............................207 "

Three hundred parts by weight of this mixture are taken to five hundred parts of gauze. Or the following may be used:

℞ Vaseline.............................. 30 parts.
Carbolic acid.......................... 28 "
Benzin.................................242 "

Three hundred for five hundred of gauze. Thymolated gauze:

℞ Thymol.............................. 10 parts.
Essence of turpentine............. 3 "
Paraffin oil........................... 10 "
Benzin.................................200 "

Three hundred and three of solution to five hundred of the gauze. Sublimated gauze:

℞ Bichloride of mercury............. $1\frac{1}{2}$ parts.

Chloride of sodium................. $\frac{1}{2}$ part.

Glycerine.............................. 15 parts.

Distilled water........................500 "

Equal parts of the solution and gauze are employed. Iodoform gauze:

℞ Iodoform.............................. 50 parts.
Paraffin oil........................... 10 "
Ether...................................400 "

The weight ratio between the amount of solution used and the gauze is four hundred and sixty to five hundred. The gauze is allowed to soak for twelve hours in this solution, is then dried, and stored in an antiseptic, air-tight jar.—*La Medecine Moderne.*

